# Correction to: Determinants of activity and efficacy of anti-PD1/PD-L1 therapy in patients with advanced solid tumors recruited in a clinical trials unit: a longitudinal prospective biomarker-based study

**DOI:** 10.1007/s00262-023-03396-5

**Published:** 2023-02-21

**Authors:** Javier García-Corbacho, Alberto Indacochea, Azucena E. González Navarro, Iván Victoria, Débora Moreno, David Pesántez, Laura Angelats, Andrea Modrego-Sanchez, Esther Sanfeliu, Oleguer Castillo, Paula Blasco, Laura Mezquita, Nuria Viñolas, Miquel Nogué, Patricia Galván, Barbara Adamo, Neus Basté, Tamara Sauri, Manel Juan, Aleix Prat, Francesco Schettini

**Affiliations:** 1 Medical Oncology Department, Hospital Clinic of Barcelona, Barcelona, Spain; 2 Medical Oncology Department (UGCI), Virgen de La Victoria and Regional University Hospital / IBIMA, Málaga, Spain; 3 Medical Oncology Department, Hospital General of Granollers, Barcelona, Spain; 4 Immunology Department, Hospital Clinic of Barcelona, Barcelona, Spain; 5 Translational Genomics and Targeted Therapies in Solid Tumours Group, August Pi i Sunyer Biomedical Research Institute (IDIBAPS), Barcelona, Spain; 6 Medical Oncology Department, University Hospital, 12 de Octubre, Madrid, Spain; 7 Pathology Department, Diagnostic Biomedical Center, Hospital Clinic of Barcelona, Barcelona, Spain; 8 Departament de Medicina, Facultat de Medicina i Ciències de la Salut, Universitat de Barcelona, c. Casanova, 143, 08036 Barcelona, Spain

**Correction to: Cancer Immunology, Immunotherapy** 10.1007/s00262-022-03360-9

The original version of this article unfortunately contained a mistake. Given name and family names were Interchanged.

The correct given names and family names should be:

Given name Javier

Family name García-Corbacho

Given name Alberto

Family name Indacochea

Given name Azucena E

Family name González Navarro

Given name Iván

Family name Victoria

Given name Débora

Family name Moreno

Given name David

Family name Pesántez

Given name Laura

Family name Angelats

Given name Andrea

Family name Modrego-Sanchez

Given name Esther

Family name Sanfeliu

Given name Oleguer

Family name Castillo

Given name Paula

Family name Blasco

Given name Laura

Family name Mezquita

Given name Nuria

Family name Viñolas

Given name Miquel

Family name Nogué

Given name Patricia

Family name Galván

Given name Barbara

Family name Adamo

Given name Neus

Family name Basté

Given name Tamara

Family name Sauri

Given name Manel

Family name Juan

Given name Aleix

Family name Prat

Given name Francesco

Family name Schettini

The original article has been corrected.

